# High Mobility Group Box 1 Protein in Cerebral Thromboemboli

**DOI:** 10.3390/ijms222011276

**Published:** 2021-10-19

**Authors:** Fabian Essig, Lilith Babilon, Christoph Vollmuth, Alexander M. Kollikowski, Mirko Pham, László Solymosi, Karl Georg Haeusler, Peter Kraft, Guido Stoll, Michael K. Schuhmann

**Affiliations:** 1 Department of Neurology, University Hospital Würzburg, 97080 Würzburg, Germany; Essig_F@ukw.de (F.E.); Babilon_L@ukw.de (L.B.); Vollmuth_C1@ukw.de (C.V.); Haeusler_K@ukw.de (K.G.H.); Peter.kraft@klinikum-msp.de (P.K.); Stoll_G@ukw.de (G.S.); 2 Department of Neuroradiology, University Hospital Würzburg, 97080 Würzburg, Germany; Kollikowsk_A@ukw.de (A.M.K.); Pham_M@ukw.de (M.P.); Solymosi_L@ukw.de (L.S.); 3 Department of Neurology, Klinikum Main-Spessart, 97816 Lohr, Germany

**Keywords:** acute ischemic stroke, thromboemboli, HMGB1, neutrophils, platelets, immunohistochemistry

## Abstract

High-mobility group box 1 protein (HMGB1) is a damage-associated molecular pattern (DAMP) involved in neutrophil extracellular trap (NET) formation and thrombosis. NETs are regularly found in cerebral thromboemboli. We here analyzed associated HMGB1 expression in human thromboemboli retrieved via mechanical thrombectomy from 37 stroke patients with large vessel occlusion. HMGB1 was detected in all thromboemboli, accounting for 1.7% (IQR 0.6–6.2%) of the total thromboemboli area and was found to be colocalized with neutrophils and NETs and in spatial proximity to platelets. Correlation analysis revealed that the detection of HMGB1 was strongly related to the number of neutrophils (r = 0.58, *p* = 0.0002) and platelets (r = 0.51, *p* = 0.001). Our results demonstrate that HMGB1 is a substantial constituent of thromboemboli causing large vessel occlusion stroke.

## 1. Introduction

High mobility group box 1 (HMGB1) is a non-histone protein, which is constitutively expressed in most cell types and can be released actively or passively into the extracellular space from necrotic or activated cells [1,2]. In cerebral ischemia, HMGB1 is mainly released by dying neurons, and circulating HMGB1 levels correlate with ischemic brain damage [3,4,5]. In addition, platelets and leukocytes are another important source of damage-associated molecular patterns (DAMPs) such as HMGB1, which has been largely neglected in the stroke field [5,6]. Platelet-derived HMGB1 is a critical mediator of thrombosis [7] and neutrophil extracellular trap (NET) formation [8]. Recently, several publications reported a significant contribution of NETosis in vessel occlusive thromboemboli retrieved during mechanical thrombectomy (MT) in acute ischemic stroke patients [9,10,11]. NETs, as extracellular, web-like structures consisting of DNA, histones and granules, are also involved in thrombosis and might increase thrombus stability and impact thrombolytic resistance [12,13]. We here analyzed whether or not cerebral thromboemboli contain HMGB1 and how HMGB1 relates to cellular components such as platelets and neutrophils.

## 2. Results

### 2.1. HMGB1 Expression in Cerebral Thromboemboli

**Figure 1:**
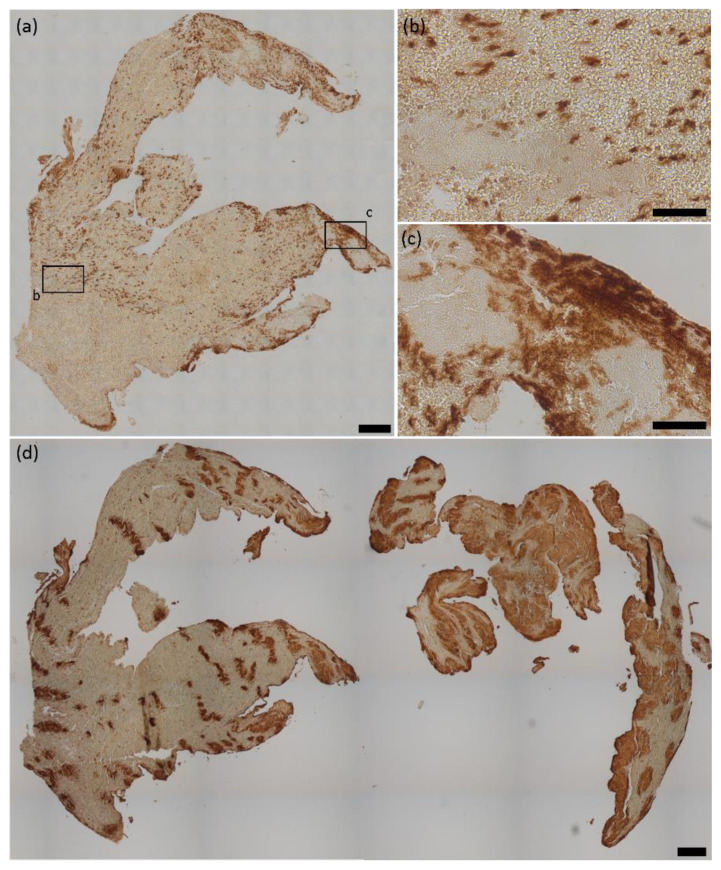
HMGB1 and platelets in cerebral thromboemboli. (**a**) Representative HMGB1 stained thromboemboli and magnifications illustrating the presence of (**b**) small, spotted HMGB1 regions, whereas, especially in peripheral regions, (**c**) extensive areas of HMGB1 were found. (**d**) Representative CD42b stained thromboemboli illustrating the diversity of platelet-rich areas within cerebral thromboemboli. HMGB1 = high mobility group box 1. Scale bars: (**a**,**d**) 200 µm, (**b**,**c**) 50 µm.

HMGB1 was expressed in all 37 thromboemboli, accounting for 1.7% (IQR 0.6–6.2%) of the total thromboemboli area (Figure 1a), as revealed by immunohistochemistry in small spotted areas within erythrocyte- and fibrin-rich areas (Figure 1b). Frequently, HMGB1 was also found to be widely distributed, especially in peripheral outer regions of the cerebral thromboemboli (Figure 1c).

Quantitative analysis of CD42b-positive areas as a measure for platelets revealed that cerebral thromboemboli contain an amount of platelet-rich material between 5.3 and 65.2% (mean 30.7 ± 14.3%) of the total thromboemboli area. Platelet rich areas are also highly variable in thrombus’ morphology and were found as curved, independent structures within large erythrocyte-rich areas but also as large and widespread regions within cerebral thromboemboli (Figure 1d).

**Figure 2:**
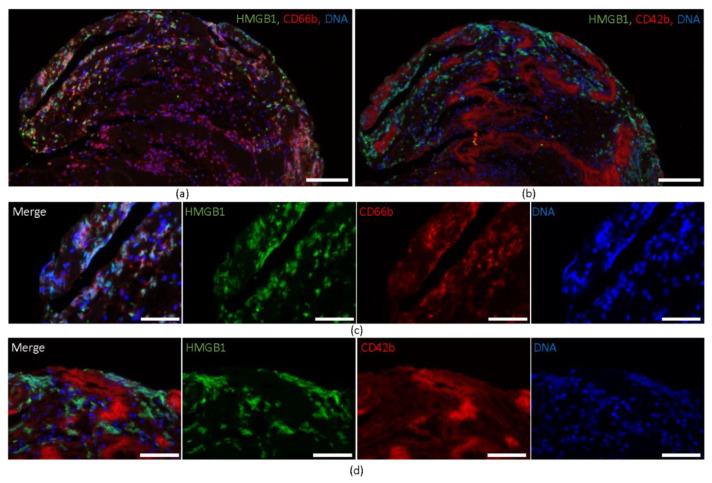
Immunofluorescent staining of HMGB1. Immunofluorescent co-staining identifies colocalization of (**a**) HMGB1, CD66b and DNA (by DAPI) and spatial proximity of (**b**) HMGB1 and CD42b. Representative magnifications illustrate the spatial coexistence of HMGB1 and CD66b, whereas CD42b was detected within the interspace of HMGB1-positive areas and has only marginal contact with HMGB1. Scale bars: (**a**,**b**) 100 µm, (**c**,**d**) 50 µm. HMGB 1 = high mobility group box 1, CD = cluster of differentiation, DAPI = 4,6-diamidino-2-phenylindole.

For cellular localization of HMGB1, triple immunofluorescence staining was performed for HMGB1, CD66b, a marker for granulocytes, and CD42b. Fluorescent co-staining showed spatial colocalization of HMGB1 and CD66b and spatial proximity of HMGB1 expression to CD42b-positive platelets, but no colocalization (Figure 2a,b). Higher magnifications confirmed the spatial coexpression of HMGB1 and DNA within CD66b-positive granulocytes (Figure 2c), whereas areas covered by CD42b-positive platelets were found frequently within the interspace of HMGB1 positive areas and had only marginal direct contact with HMGB1 (Figure 2d).

**Figure 3:**
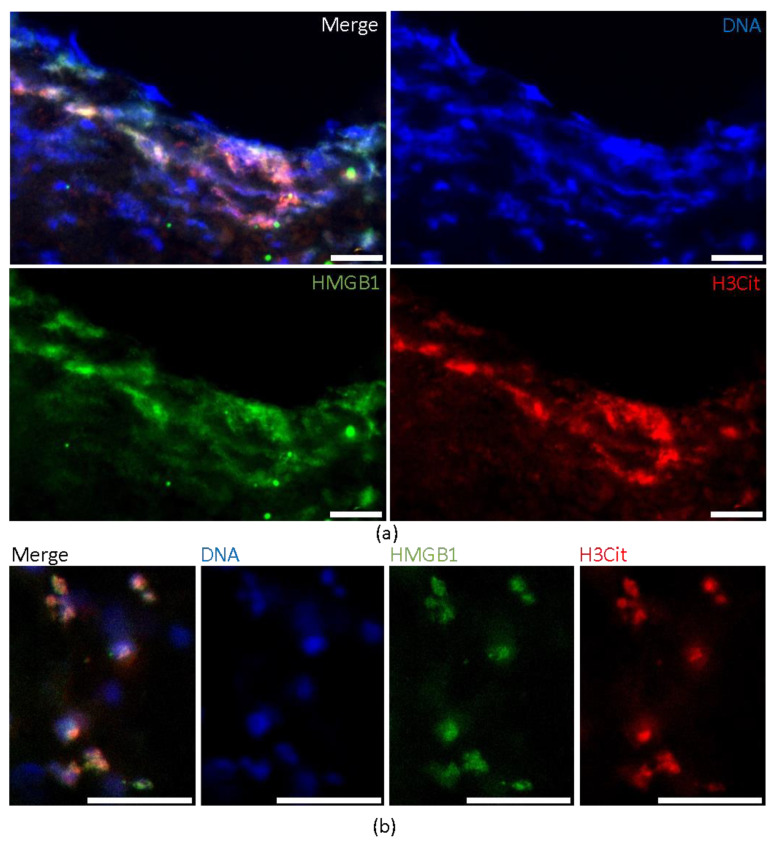
Immunofluorescent coexistence of HMGB1 and NETs. Immunofluorescent co-staining identifies large areas with colocalization of (**a**) DNA (by DAPI), HMGB1 and H3Cit demonstrating the existence of HMGB1 in NETs. Representative magnifications illustrate small punctual coexistence (**b**) of HMGB1 and H3Cit revealing NETosing neutrophils. Scale bars: (**a**,**b**) 20 µm. HMGB1 = high mobility group box 1, DAPI = 4,6-diamidino-2-phenylindole.

Additional fluorescent co-staining of HMGB1 and H3Cit revealed a spatial co-expression of DNA, HMGB1 and H3Cit within large areas of NETs (Figure 3a), but also dotted areas, implying NETosing neutrophils (Figure 3b).

### 2.2. HMGB1 Correlates with the Amount of Neutrophils and Platelets

**Figure 4:**
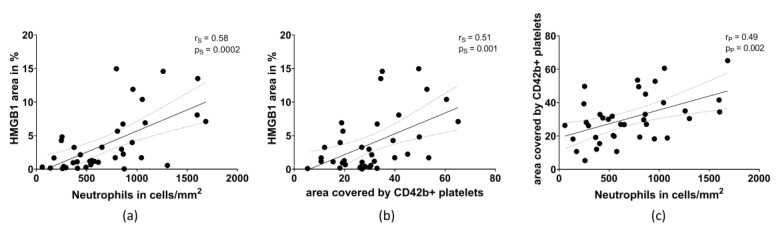
Correlation between (**a**) the area of HMGB1 (in % of the total thromboembolus area) and the amount of neutrophils, (**b**) the area of HMGB1 and the area covered by CD42b-positive platelets, and (**c**) the amount of neutrophils with the area covered by CD42b-positive platelets. *p* = level of significance. r = correlation coefficient. s = Spearman, p = Pearson.

Quantitative analysis of HMGB1-positive areas, areas covered by CD42b-positive platelets and the number of neutrophils per mm^2^ were performed for the whole thromboembolus area. Correlation analysis of HMGB1 and structural components of cerebral thromboemboli was performed: the amount of HMGB1 strongly correlated with the number of neutrophils (r = 0.58, *p* = 0.0002) (Figure 4a). A positive correlation was also found for HMGB1-positive area and the area covered by CD42b-positive platelets (r = 0.51, *p* = 0.001) (Figure 4b). Furthermore, the number of neutrophils was also associated with the area covered by CD42b-positive platelets (r = 0.49, *p* = 0.002) (Figure 4c).

## 3. Discussion

Cerebral thromboemboli causing large vessel occlusion and subsequent ischemic stroke exhibit a high structural complexity involving inflammatory cells [10,14,15]. HMGB1 is an endogenous DAMP-molecule, which can induce inflammation, but also thrombus formation [16,17]. As the principal finding, we demonstrate that HMGB1 is strongly expressed in human cerebral thromboemboli, further linking inflammation and thrombus formation. HMGB1 is colocalized with NETosing neutrophils and large areas of extruded NETs, which are regularly seen in cerebral thromboemboli [9,10,11]. CD42b-immunoreactivity-positive platelets were in close vicinity to HMGB1-positive neutrophils and NETs, but did not express HMGB1 although they represent a major source of HMGB1. It is well established that platelets release HMGB1 into the extracellular space upon activation [6,18]. Secreted HMGB1 can autoactivate platelets on the surface and thereby contribute to platelet aggregation and thrombus formation [7,19]. More importantly, platelet-derived HMGB1 can induce autophagy in neutrophils and formation of NETs [6,8]. NETosis itself augments inflammation and has prothrombotic effects by activating platelets [20]. Thereby a vicious cycle of activation and reactivation between platelets and neutrophils can emerge since HMGB1 can also be released by NETosed neutrophils [6]. The strong colocalization of HMGB1 and NETs in cerebral thromboemboli, together with the recent finding of a local increase in HMGB1 plasma levels related to the extent of neutrophil recruitment in the ischemic cerebral circulation of stroke patients [5], supports the notion that extracellular HMGB1 derived from activated platelets and NETosed neutrophils is an early mediator in thrombus growth and cerebral embolism.

Taken together, our study identifies HMGB1 as an important structural component in human cerebral thromboemboli and supports the notion that not only platelet aggregation but also platelet-driven inflammation are an integral part of arterial thrombus formation.

## 4. Materials and Methods

### 4.1. Patient Population and Thrombectomy Procedure

For immunohistochemical analysis, 37 human thromboemboli causing acute ischemic stroke were retrieved during mechanical thrombectomy at the Department of Neuroradiology, University Hospital of Würzburg, Germany, as previously described [10,14]. The ethics committee of the Medical Faculty of the University of Würzburg approved the study protocol (reference number 36/12, 13 March 2012). Mechanical thrombectomy was performed according to local standards using stent retrievers. Patients received general anesthesia during the intervention.

### 4.2. Immunohistochemistry and Quantification

Thromboembolus processing after retrieval and immunohistochemical staining protocols were performed as described previously [10,14]. Immunohistological staining was performed for HMGB1, CD42b and CD66b. Primary antibodies (mouse anti-human HMGB1 polyclonal antibody (1:50, Ab190377, Abcam, Cambridge, UK), rabbit anti-human CD66b polyclonal antibody (1:50, Ab197678, Abcam, Cambridge, UK), mouse anti-human CD42b polyclonal antibody (1:100, MA5-11642, Invitrogen, Carlsbad, CA, USA) were used. Immunofluorescence staining protocol was performed as explained earlier [10]. As primary antibodies, mouse anti-human HMGB1 polyclonal antibody (1:50, Ab190377, Abcam, Cambridge, UK), rabbit anti-human CD42b antibody (1:100, ab183345, Abcam, Cambridge, UK), rabbit anti-human CD66b polyclonal antibody (1:50, Ab197678, Abcam, Cambridge, UK) and rabbit anti-human H3Cit antibody (1:50, ab 5103, Abcam, Cambridge, UK) were applied. As secondary antibodies (Alexa Fluor 488 goat anti-mouse IgG (1:100, A11001, Invitrogen, Carlsbad, CA, USA) and Alexa Fluor 594 goat anti-rabbit IgG (1:100 A21244, Invitrogen, Carlsbad, CA, USA) were incubated. Leica DMi8 and LasX software was used for image acquisition. The HMGB1-positive area and CD42-positive area was determined by Image J software (https://imagej.nih.gov/ij/ (accessed on 13 August 2019)) in percentage of the total thromboembolus area. Quantification of neutrophils was performed as already described [10].

### 4.3. Quantification and Statistical Analysis

Statistical analysis was performed with Graph Pad Prism 8.0.2 (Graph Pad Software, San Diego, CA, USA). Normal distribution was tested by means of the Kolmogorov–Smirnov normality test. Pearson/Spearman correlations were performed to test for significance. *p* values < 0.05 were considered to indicate statistical significance.

## Data Availability

The data presented in this study are available on reasonable request from the corresponding author. The data are not publicly available due to privacy and ethical restrictions.
